# Multiscale modeling of cortical information flow in Parkinson's disease

**DOI:** 10.1186/1471-2202-14-S1-O21

**Published:** 2013-07-08

**Authors:** Cliff C Kerr, Sacha J van Albada, Samuel A Neymotin, George L Chadderdon III, Peter A Robinson, William W Lytton

**Affiliations:** 1 Department of Physiology and Pharmacology, SUNY Downstate Medical Center, Brooklyn, NY, USA; 2 Complex Systems Group, School of Physics, University of Sydney, NSW, Australia; 3 Institute of Neuroscience and Medicine (INM-6) and Institute for Advanced Simulation (IAS-6), Jülich Research Centre and JARA, Jülich, Germany; 4 Department of Neurobiology, Yale University, New Haven, CT, USA

## 

The basal ganglia play a crucial role in the execution of movements, as demonstrated by the severe motor deficits that accompany the neuronal degeneration underlying Parkinson's disease (PD). Since motor commands originate from the cortex, an important functional question is how the basal ganglia influence cortical information flow, and how this influence becomes pathological in PD. In contrast, previous models of PD have focused on either global brain dynamics or on local information flow, since developing a model that is valid on both scales presents a major technical challenge.

To address this issue, we developed a composite neuronal network/neural field model. The neuronal network consisted of 4950 event-driven rule-based neurons, divided into 15 excitatory and inhibitory cell populations in the thalamus and cortex. This model was then embedded in a neural field model of the basal ganglia-thalamocortical system, including the cortex, thalamus, striatum, subthalamic nucleus, and globus pallidus. Both network and field models have been separately validated in previous work [1-3], with both shown to produce realistic firing rates and spectra. Two field models were explored: one with parameters based on data from healthy individuals, and one based on data from individuals with PD. Spikes generated by these field models (which represent inputs from distant brain areas) were then used to drive the network model (which represents a small region of association cortex). We then explored the effects that these drives had on the information flow and dynamics of the network.

**Figure 1:**
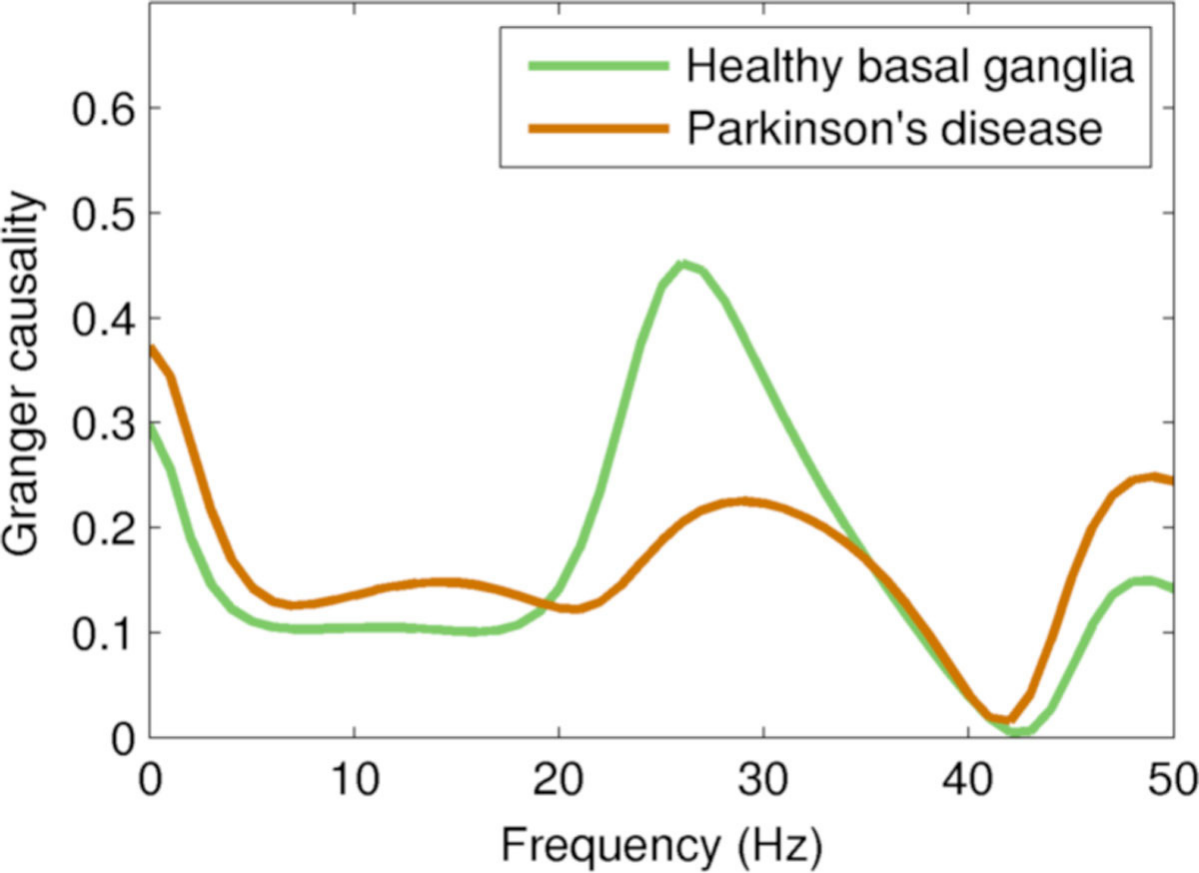
**Spectral Granger causality from layer 4 to layer 5, a major feedforward pathway in the cortex**. The healthy model shows strong causality in the high-beta/low-gamma (20-35 Hz) band; this is almost entirely lost in the PD-driven model, resulting in substantially lower total causality.

Compared to the network driven by the healthy field model, the PD-driven network had lower firing rates and increased power at low frequencies, consistent with clinical PET and EEG findings; it also had more spike bursts, indicating pathologically increased intracortical coherence. The PD-driven network showed significant reductions in Granger causality (see Figure 1). In particular, the reduction in Granger causality from the main "input" layer of the cortex (layer 4) to the main "output" layer (layer 5) represents a possible explanation for some of the characteristics of parkinsonism, such as bradykinesia. These results demonstrate that the brain's large-scale oscillatory environment, represented here by the field model, strongly influences the information processing that occurs within its subnetworks.
